# Combination of 35-Gene Mutation Profile and Radiotherapy Dosimetry Predicts the Therapeutic Outcome of Definitive Chemoradiation in Patients With Esophageal Squamous Cell Carcinoma

**DOI:** 10.3389/fonc.2021.729418

**Published:** 2021-08-27

**Authors:** Peng Tang, Chen Tan, Qingsong Pang, Chih-Wen Chi, Yuwen Wang, Zhiyong Yuan, Yu-Chuen Huang, Yu-Jen Chen

**Affiliations:** ^1^Department of Esophagus Surgery, Key Laboratory of Prevention and Therapy, National Clinical Research Center of Cancer, Tianjin Medical University Cancer Institute and Hospital, Tianjin, China; ^2^Department of Radiation Oncology, Minzu Hospital of Guangxi Zhuang Autonomous Region, Affiliated Minzu Hospital of Guangxi Medical University, Nanning, China; ^3^Department Radiation Oncology, Tianjin Medical University Cancer Institute and Hospital, National Clinical Research Center for Cancer, Key Laboratory of Cancer Prevention and Therapy, Tianjin’s Clinical Research Center for Cancer, Tianjin, China; ^4^Department of Medical Research, MacKay Memorial Hospital, New Taipei City, Taiwan; ^5^School of Chinese Medicine, China Medical University, Taichung, Taiwan; ^6^Department of Medical Research, China Medical University Hospital, Taichung, Taiwan; ^7^Department of Radiation Oncology, MacKay Memorial Hospital, Taipei, Taiwan; ^8^Department of Nursing, MacKay Junior College of Medicine, Nursing and Management, Taipei, Taiwan

**Keywords:** lung radiation dose, concurrent chemoradiotherapy, 35-gene panel, squamous cell carcinoma, esophageal cancer

## Abstract

Esophageal cancer is a common malignancy worldwide and a leading cause of cancer-related mortality. Definitive concurrent chemoradiotherapy (CCRT) has been widely used to treat locally advanced esophageal squamous cell carcinoma (ESCC). In this study, we evaluated the predictive power of a 35-gene mutation profile and radiation parameters in patients with ESCC. Data from 44 patients with ESCC who underwent definitive CCRT were retrospectively reviewed. A 35-gene mutation profile, derived from reported ESCC-specific next-generation sequencing results, and radiation dosimetry parameters were examined using the Kaplan–Meier curve and Cox proportional hazards model. All patients were native Chinese and underwent CCRT with a median follow-up time of 22.0 months. Significant prognostic factors affecting progression-free survival in the multivariable Cox regression model were clinical nodal staging ≥2 (hazard ratio, HR: 2.52, 95% CI: 1.15–5.54, *p* = 0.022), ≥10% lung volume receiving ≥30 Gy (V30) (HR: 2.36, 95% CI: 1.08–5.17, *p* = 0.032), and mutation of fibrous sheath interacting protein 2 (*FSIP2*) (HR: 0.08, 95% CI: 0.01–0.58, *p* = 0.013). For overall survival, significant prognostic factors in the multivariable Cox regression model were lung V30 ≥10% (HR: 3.71, 95% CI: 1.48–9.35, *p* = 0.005) and mutation of spectrin repeat containing nuclear envelope protein 1 (*SYNE1*) (HR: 2.95, 95% CI: 1.25–6.97, *p* = 0.014). Our cohort showed higher *MUC17* (79.5% vs. 5.7%), *FSIP2* (18.2% vs. 6.2%), and *SYNE1* (38.6% vs. 11.0%) mutation rates and lower *TP53* (38.6% vs. 68.7%) mutation rates than the ESCC cohorts from The Cancer Genome Atlas. In conclusion, by using a combination of a 35-gene mutation profile and radiotherapy dosimetry, mutations in *FSIP2* and *SYNE1* as well as lung V30 were identified as potential predictors for developing a prediction model for clinical outcomes in patients with ESCC administered definitive CCRT.

## Introduction

Esophageal cancer is the sixth and seventh most common cause of cancer-related mortality and malignancy worldwide, respectively (1). Esophageal adenocarcinoma and esophageal squamous cell carcinoma (ESCC) are the major histopathological types of esophageal cancer, and ESCC is common in Eastern and Central Asia (2). Given the difficulty of early screening for ESCC, most patients are diagnosed at a locally advanced stage.

Among the treatment modalities for esophageal cancer, concurrent chemoradiotherapy (CCRT), as a definitive or neoadjuvant therapy, is beneficial for improving the disease prognosis (3–5). Definitive CCRT and neoadjuvant CCRT followed by surgery are recommended for treating unresectable or locally advanced esophageal cancer, including ESCC. For locally advanced ESCC, the effectiveness of neoadjuvant CCRT on improving survival have been demonstrated in NEOCRTEC 5010 trial (focusing on ESCC) and CROSS trial (with 23% ESCC patients) (6, 7). The definitive CCRT for locally advanced ESCC with response to initial chemoradiation has similar clinical outcome in comparison to initial chemoradiation followed by surgery (8). Taken together, both definitive and neoadjuvant CCRT are optional treatment modalities for locally advanced ESCC. However, long-term survival rate of patients with locally advanced ESCC remains less than 30% (9). Therefore, the development of informative predictors of the prognosis of patients with ESCC is clinically important.

In recent years, advances in technologies for high-throughput genomic surveys, including next-generation sequencing (NGS) of DNA, have enabled comprehensive characterization of somatic mutations in clinical specimens. Through whole-exome or whole-genome sequencing, differential mutations in matched DNA between normal and ESCC tissues have been identified. Among the reported mutations, those in *TP53*, *CDKN2A*, *FAT1*, *NOTCH1*, *PIK3CA*, *KMT2D*, and *NFE2L2* were validated as candidate biomarkers for ESCC development. However, there are no biomarkers for predicting the clinical outcomes of ESCC treatment. Therefore, in this study, we evaluated the predictive power of a 35-gene mutation profile, clinicopathological characteristics of patients with ESCC, and radiation parameters in assessing the clinical outcomes in patients with ESCC treated with CCRT.

## Materials and Methods

### Patients

Between 2014 and 2017, data for 44 patients with ESCC who received definitive CCRT were retrospectively reviewed. All patients were diagnosed with ESCC by pathological examination of biopsied specimens. The patient characteristics are shown in **Table 1**. As the incidence rate of ESCC is higher in males than in females (2), our study population showed sex inequality. We evaluated the distribution of patient age at diagnosis (male: 22.7 ± 13.9 years; female: 21.2 ± 13.0 years, *t*-test: *p* > 0.05), follow-up period (median period, male: 24.4 months; female: 20.1 months, Mann-Whitney U test: *p* > 0.05), and clinical T and N stages (χ^2^ test, *p* > 0.05), which did not significantly differ between males and females. All seven female patients received upfront CCRT, and 56.8% of male patients received upfront CCRT; 43.2% of male patients received upfront CCRT followed by adjuvant therapy. The study was approved by the Ethics Committee of Tianjin Medical University Institute and Hospital (documentation number #bc2018057). Written informed consent was obtained from all study participants.

**Table 1:** Characteristics and pathological features of esophageal cancer patients.

Characteristics	Total (%) (n = 44)
Sex (male/female)	
Male	37 (84.1%)
Female	7 (15.6%)
Age of diagnosis (mean ± SD; range)	61.0 ± 5.2; 50–69 years
Follow up period (median, min-max)	22.0, 3.2–50.1 months
ECOG performance status	
0	0 (0%)
1	44 (100%)
Clinical stage	
T stage	
T1	2 (4.5%)
T2	1 (2.3%)
T3	29 (65.9%)
T4	12 (27.3%)
N stage	
N0	7 (15.9%)
N1	16 (36.4%)
N2	17 (39.6%)
N3	4 (9.1%)
M stage	
M0	44 (100%)
M1	0 (0%)
Upfront chemotherapeutic regimen	44 (100%)
paclitaxel plus cisplatin	44 (100%)
Adjuvant chemotherapeutic regimen	16 (36.4%)
paclitaxel plus cisplatin	15 (93.8%)
paclitaxel plus cisplatin + XELOX	1 (6.2%)

ECOG, Eastern Cooperative Oncology Group.

### Concurrent Chemoradiation

**Figure 1:**
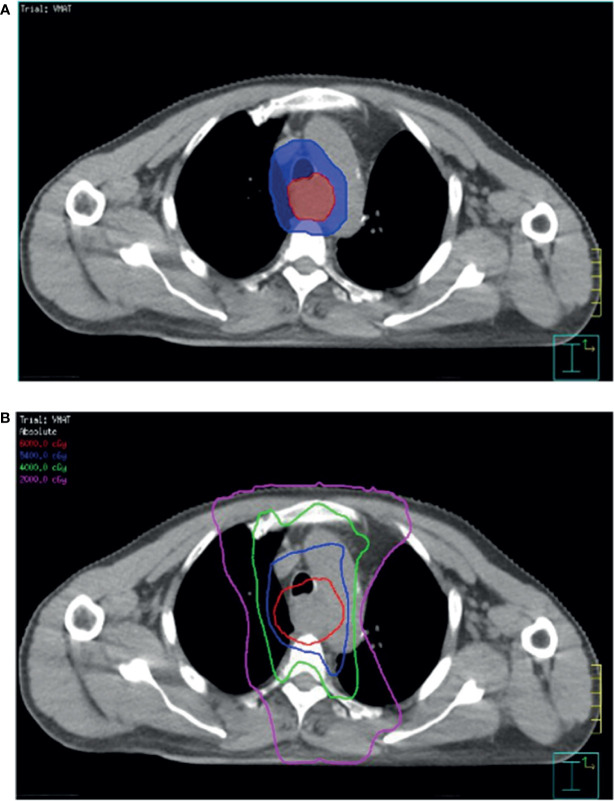
Computed tomography simulation image of radiotherapy planning. **(A)** Simultaneous integrated boost intensity-modulated radiotherapy used in patients with ESCC. **(B)** Planned target volume (blue area) is an expansion of the gross tumor volume (red area). The isodose lines represent the total doses of 60 Gy (red), 54 Gy (blue), 40 Gy (green), and 20 Gy (pink).

The prescribed radiation dose was 60 Gy in 30 fractions for the gross tumor and 54 -60 Gy in 30 fractions for the regional lymphatics (**Figure 1**). All patients underwent concurrent chemotherapy during radiotherapy (RT). RT was planned based on simulation computed tomography (CT) images. The patients were immobilized in a supine position using an Alpha Cradle^®^ (Smithers Medical Products, Inc., North Canton, OH, USA), and simulation CT scan images (Brilliance Big Bore CT simulator/Philips Medical Systems, Cleveland, OH, USA) were acquired at a slice thickness of 3 mm. The gross tumor volume was determined for the esophageal gross tumor, and a margin extension of 0.5–1.0 cm was considered the clinical target volume for enlarged regional lymph nodes. The planning target volume enclosed the clinical target volume with margins based on institutional assessment to account for uncertainties in the set-up or internal organ motion. Intensity-modulated RT with the simultaneous integrated boost technique was delivered to the planning target volume, as shown in **Figure 1**. Normal organ constraints were applied to limit the total lung from receiving >20 Gy (V20) to 20% (lung V20 <20%). The maximal dose to the spinal cord was limited to <45 Gy. Treatment was optimized to ensure that at least 95% of the planning target volume was covered by the prescribed dose. RT was withheld for patients showing a ≥grade 3 reduction in the neutrophil or platelet count (absolute neutrophil count <1000 cells/μL or platelet count <50,000 cells/μL). The chemotherapeutic regimen in the study was paclitaxel plus cisplatin either in front or adjuvant chemotherapy, except for in one patient with clinical stage IV who was administered paclitaxel plus cisplatin and XELOX as an adjuvant therapy. All 44 patients were administered front chemotherapy and 36.4% (16/44) patients underwent CCRT followed by adjuvant chemotherapy. There was no difference in the characteristics of patients in the front and adjuvant chemotherapy groups. Chemotherapy was delayed if ≥grade 2 toxicities developed (absolute neutrophil count <1500 cells/μL or platelet count <75,000 cells/μL). The cisplatin dose was adjusted according to the renal function of the patients.

### Genomic DNA Isolation

For each specimen, genomic DNA from formalin-fixed paraffin-embedded tissue was extracted using a Cobas^®^ DNA Sample Preparation Kit (Roche, Basel, Switzerland) according to the manufacturer’s protocol. DNA was quantified using a Qubit^®^ dsDNA HS Assay Kit (Thermo Fisher Scientific, Waltham, MA, USA). A High Sensitivity DNA Kit (Agilent Technologies, Santa Clara, CA, USA) was used for quality control, and the fragment distribution was analyzed using a 2100 Bioanalyzer (Agilent Technologies).

### DNA Library Construction and NGS

DNA (10 ng) from all 44 samples was used to construct amplicon-specific DNA libraries. A custom ESCC panel comprising 35 genes, 159 amplicons, and over 275 hotspots, developed by Lihpao (Xiamen) Biotechnology Co., Ltd. (China, Fujian), was used. The following genes are included in the panel: *TNN*, *HMCN1*, *USH2A*, *LRP1B*, *XIRP2*, *LRP2*, *NFE2L2*, *TTN*, *FSIP2*, *SI*, *PIK3CA*, *MUC4*, *FBXW7*, *FAT1*, *DNAH5*, *TRIO*, *GPR98*, *SYNE1*, *ABCA13*, *PCLO*, *MUC17*, *ZFHX4*, *CSMD3*, *CDKN2A*, *NOTCH1*, *MUC2*, *FAT3*, *KMT2D*, *RB1*, *TP53*, *MYH4*, *MUC16*, *EP300*, *DMD*, and *KDM6A*. A DNA library was generated using Ion AmpliSeq Library Kit 2.0 (Thermo Fisher Scientific) according to the manufacturer’s protocol. The quantified libraries were clonally amplified on ion sphere particles by emulsion polymerase chain reaction using the Ion OneTouch™ 2 system with the Ion PGM Hi-Q View OT2 Kit (Thermo Fisher Scientific). Next, the ion sphere particles were enriched in an Ion OneTouch™ ES instrument (Thermo Fisher Scientific). Finally, the enriched ion sphere particles were loaded onto the 316 chip, and sequencing was performed on an Ion Torrent PGM system (Ion Torrent, Paisley, UK) using an Ion PGM Hi-Q View Sequencing Kit (Thermo Fisher Scientific).

### Data Analysis

The personal genome machine-based DNA sequencing data were generated using Torrent Suite software (Thermo Fisher Scientific). Variant calling and annotation were conducted using Ion-Reporter v5.1.0. Mutations with an average coverage of ≥1500 reads and a mutant allele frequency of ≥5% were reported. The original contributions presented in the study are publicly from https://www.ncbi.nlm.nih.gov/sra/PRJNA742478. To further explore the distinct variations observed in our study, we compared our data with those of ESCC cohorts obtained from The Cancer Genome Atlas (TCGA) *via* cBioPortal (http://www.cbioportal.org). Genomic data types integrated with cBioPortal included somatic mutations, DNA copy number alterations, mRNA and microRNA expression, and DNA methylation.

### Statistics Analysis

A 35-gene mutation profile, derived from reported ESCC-specific NGS results, and radiation dosimetry parameters were examined. PFS and OS were analyzed using Kaplan–Meier curves and the log-rank test, respectively. PFS was calculated from the time between the date of the initial biopsy and diagnosis to disease progression, relapse, or death from any cause. OS was defined as the time from the initial biopsy to the date of death. A Cox proportional hazards model was used to estimate the hazard ratios (HRs). Chi-square test or Fisher’s exact test was used to compare the gene mutation rate between the present ESCC cohort and TCGA data. All analyses were performed using SPSS Statistics v22.0 software (SPSS, Inc., Chicago, IL, USA). Results with *p*-value less than 0.05 were considered statistically significant.

## Results

### Correlation of Gene Mutations, Clinicopathological Factors, and RT Dosimetry Parameters With Clinical Outcome

**Figure 2:**
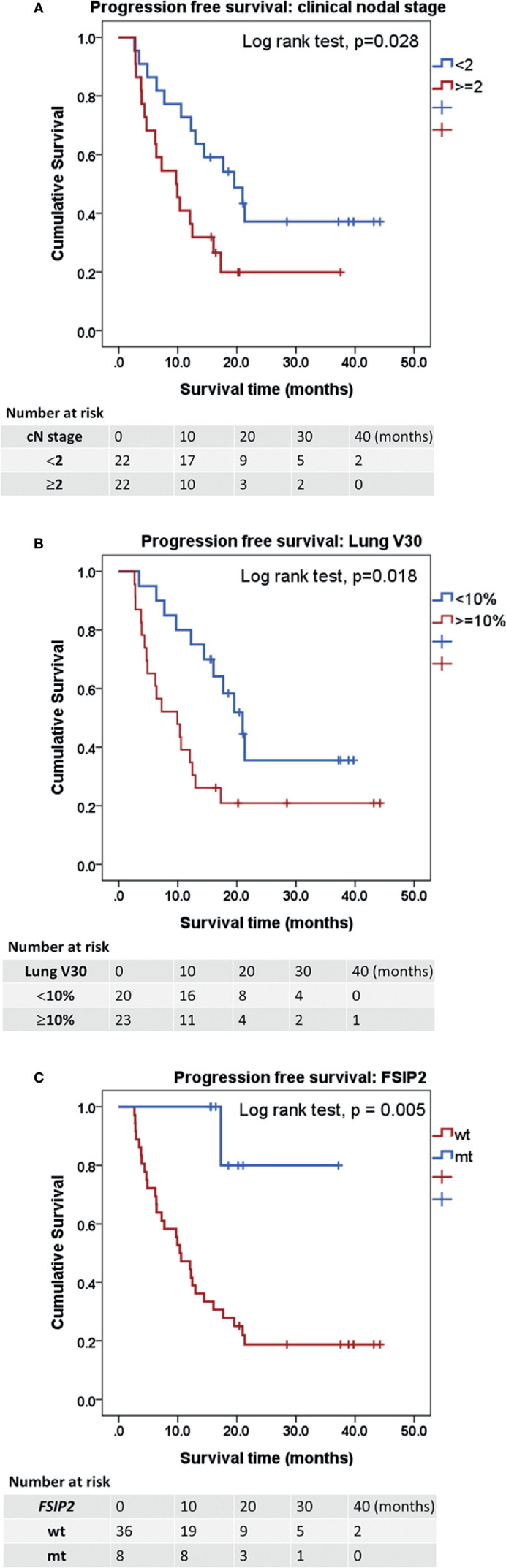
Kaplan-Meier estimates of progression-free survival. **(A)** Kaplan-Meier curves for PFS according to the factor of clinical nodal staging ≥2. *p* = 0.028. **(B)** PFS according to the factor of ≥10% lung volume receiving ≥30 Gy (V30). *p* = 0.018. **(C)** PFS according to the mutation of fibrous sheath interacting protein 2 (FSIP2). *p* = 0.005. Statistical significance was determined by Log rank test. PFS, progression free survival.

All 44 patients with ESCC were native Chinese and received CCRT with a median follow-up time of 22.0 (min–max: 3.2–50.1) months. There were no significant differences in sex, age at diagnosis, T stage, and N stage between patients at median follow-up times of <22.0 and ≥22.0 months. Univariable Cox regression analyses revealed clinical nodal staging ≥2 (HR: 2.26, 95% CI: 1.07–4.77, *p* = 0.032), ≥10% lung volume receiving ≥30 Gy (V30) (HR: 2.44, 95% CI: 1.14–5.22, *p* = 0.021), and mutation of fibrous sheath interacting protein 2 (*FSIP2*) (HR: 0.10, 95% CI: 0.01–0.72, *p* = 0.023) as significant prognostic factors for PFS. In multivariable Cox regression analyses, clinical nodal staging ≥2 (HR: 2.52, 95% CI: 1.15–5.54, *p* = 0.022), lung V30 ≥10% (HR: 2.36, 95% CI: 1.01–5.17, *p* = 0.032), and mutation of *FSIP2* (HR: 0.08, 95% CI: 0.01–0.58, *p* = 0.013) were identified as prognostic factors for PFS (**Table 2**). *FSIP2* mutation was considered as an independent factor for longer PFS. The median PFS periods of patients with clinical nodal staging ≥2 or staging <2 were 9.72 and 19.52 months (log-rank test, *p* = 0.028, **Figure 2A**), respectively. The median PFS periods of patients with lung V30 ≥10% or <10% were 9.92 and 20.96 months (*p* = 0.018, **Figure 2B**), respectively. The median PFS period of patients without the *FSIP2* mutation was 10.35 months. However, the PFS of 80% of patients with the *FSIP2* mutation was still 37.29 months (*p* = 0.005, **Figure 2C**).

**Table 2:** Cox regression analysis for progression free survival of esophageal cancer patients.

Variable	Univariable analyses		Multivariable analysis	
	HR (95% CI)	*p* value	HR (95% CI)	*p* value
cT stage (4 vs. < 4*)	0.72 (0.31-1.68)	0.441	–	–
cN stage (≥ 2 vs. < 2*)	2.26 (1.07-4.77)	*0.032*	2.52 (1.15-5.54)	*0.022*
LungV30 (≥ 10% vs. < 10%*)	2.44 (1.14-5.22)	*0.021*	2.36 (1.08-5.17)	*0.032*
FSIP2 (mt vs. wt*)	0.10 (0.01-0.72)	*0.023*	0.08 (0.01-0.58)	*0.013*
SYNE1 (mt vs. wt*)	1.50 (0.73-3.09)	0.275	–	–

*Represent reference group; HR, hazard ratio; FSIP2, fibrous sheath interacting protein 2; SYNE1, spectrin repeat containing nuclear envelope protein 1; mt, mutated; wt, wild type.

**Figure 3:**
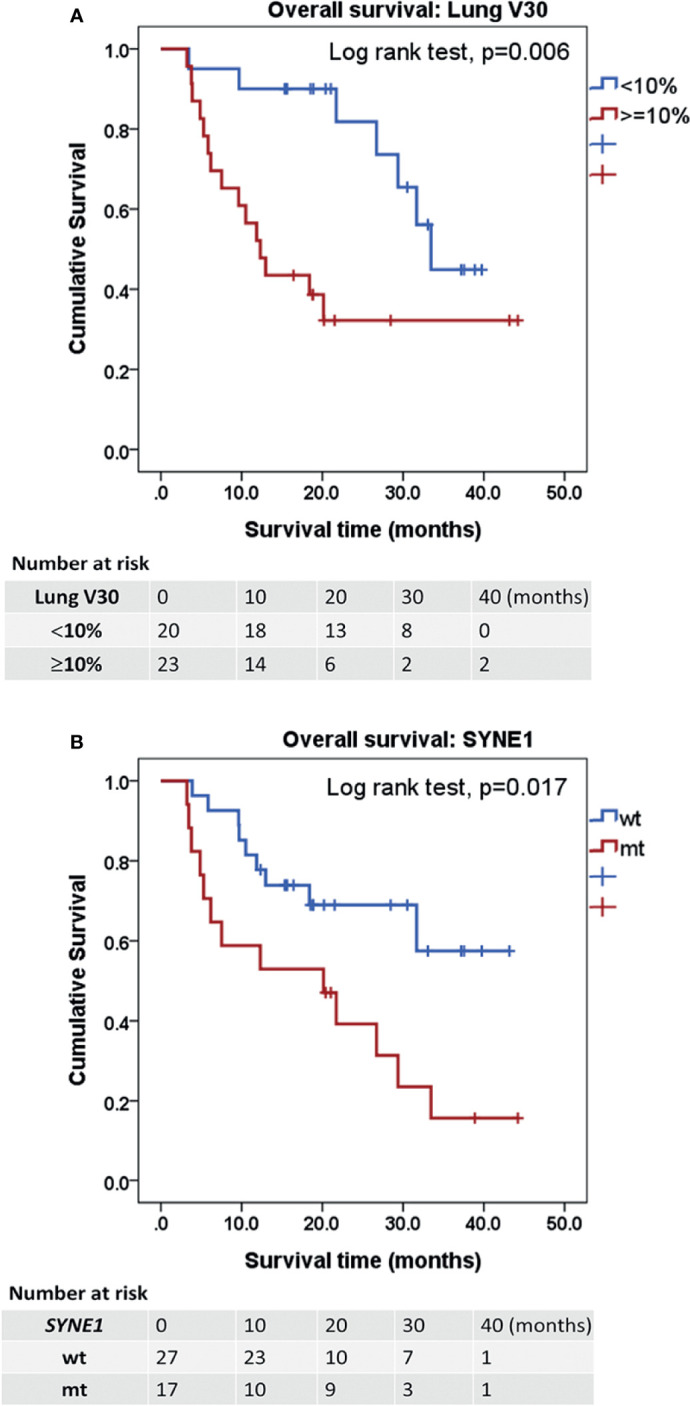
Kaplan-Meier estimates of overall survival. **(A)** Kaplan-Meier curves for OS according to the factor of ≥10% lung volume receiving ≥30 Gy (V30). *p* = 0.006. **(B)** OS according to the mutation of spectrin repeat containing nuclear envelope protein 1 (SYNE1). *p* = 0.017. Statistical significance was determined by Log rank test. OS, overall survival.

For OS, univariable Cox regression analyses revealed lung V30 ≥10% (HR: 3.40, 95% CI: 1.35–8.57, *p* = 0.009) and mutation of spectrin repeat containing nuclear envelope protein 1 (*SYNE1*) (HR: 2.71, 95% CI: 1.15–6.36, *p* = 0.022) as significant prognostic factors. In multivariable Cox regression analyses, lung V30 ≥10% (HR: 3.71, 95% CI: 1.48–9.35, *p* = 0.005) and mutation of *SYNE1* (HR: 2.95, 95% CI: 1.25–6.97, *p* = 0.014) were prognostic factors for OS (**Table 3**). *SYNE1* mutation was considered as an independent factor for worse OS. In addition, the median OS periods of patients with lung V30 ≥10% or <10% were 12.32 and 33.45 months (*p* = 0.006, **Figure 3A**), respectively. The median OS period of patients with *SYNE1* mutation was 20.14 months. More than 50% of patients without the *SYNE1* mutation were still alive at 43.17 months (*p* = 0.005, **Figure 3B**).

**Table 3:** Cox regression analysis for overall survival of esophageal cancer patients.

Variable	Univariable analyses		Multivariable analysis	
	HR (95% CI)	*p* value	HR (95% CI)	*p* value
cT stage (4 vs. < 4*)	0.58 (0.19-1.74)	0.330	–	–
cN stage (≥ 2 vs. < 2*)	1.68 (0.70-4.01)	0.245	–	–
LungV30 (≥ 10% vs. < 10%*)	3.40 (1.35-8.57)	*0.009*	3.71 (1.48-9.35)	*0.005*
FSIP2 (mt vs. wt*)	0.04 (0.00-3.57)	0.155	–	–
SYNE1 (mt vs. wt*)	2.71 (1.15-6.36)	*0.022*	2.95 (1.25-6.97)	*0.014*

*Represent reference group; HR, hazard ratio; FSIP2, fibrous sheath interacting protein 2; SYNE1, spectrin repeat containing nuclear envelope protein 1; mt, mutated; wt, wild type.

### Comparison of the Present ESCC Cohort Data With TCGA Data

Compared with the ESCC cohort data from TCGA, the data in our study showed a distinct pattern of mutation rates (**Table 4**). Significantly higher *CSMD3* (38.6% vs. 9.7%, *p* < 0.001), *DMD* (20.5% vs. 8.4%, *p* = 0.027), *EP300* (20.5% vs. 6.2%, *p* = 0.005), *FAT1* (54.5% vs. 8.8%, *p* < 0.001), *FSIP2* (18.2% vs. 6.2%, *p* ≤ 0.001), *MUC16* (47.7% vs. 14.1%, *p* < 0.001), *MUC17* (79.5% vs. 5.7%, *p* < 0.001), *NOTCH1* (31.8% vs. 8.4%, *p* < 0.001), *PIK3CA* (18.2% vs. 6.6%, *p* = 0.019), *RB1* (18.2% vs. 7.9%, *p* = 0.048), *SI* (20.5% vs. 4.8%, *p* = 0.002), *SYNE1* (38.6% vs. 11.0%, *p* < 0.001), *TTN* (61.4% vs. 33.5%, *p* < 0.001), *USH2A* (20.5% vs. 7.0%, *p* = 0.01), and *XIRP2* (29.5% vs. 7.9%, *p* < 0.001) mutation rates and a lower *TP53* (38.6% vs. 68.7%, *p* < 0.001) mutation rate were observed in our ESCC cohort compared to in the ESCC cohort from TCGA.

**Table 4:** Comparison of gene mutation rates in esophageal squamous cell carcinoma patients of the present China study group and The Cancer Genome Atlas (TCGA) cohorts.

Gene	China patients (%)	TCGA two cohorts (%)	*p* value*
*MUC17*	79.5	5.7	*<0.001* ^†^
*TTN*	61.4	33.5	*<0.001* ^†^
*FAT1*	54.5	8.8	*<0.001* ^†^
*MUC16*	47.7	14.1	*<0.001* ^†^
*CSMD3*	38.6	9.7	*<0.001* ^†^
*SYNE1*	38.6	11.0	*<0.001* ^†^
*TP53*	38.6	68.7	*<0.001* ^†^
*NOTCH1*	31.8	8.4	*<0.001* ^†^
*XIRP2*	29.5	7.9	*<0.001* ^†^
*DMD*	20.5	8.4	*0.027*
*EP300*	20.5	6.2	*0.005*
*SI*	20.5	4.8	*0.002*
*USH2A*	20.5	7.0	*0.01*
*FSIP2*	18.2	6.2	*0.014*
*PIK3CA*	18.2	6.6	*0.019*
*RB1*	18.2	7.9	*0.048*
*PCLO*	15.9	11.5	0.408^†^
*LRP1B*	13.6	11.0	0.617^†^
*ADGRV1*	11.4	9.3	0.587
*CDKN2A*	9.1	3.5	0.111
*DNAH5*	9.1	6.6	0.524
*HMCN1*	9.1	5.7	0.492
*KMT2D*	9.1	12.8	0.494^†^
*LRP2*	9.1	6.6	0.524
*ABCA13*	6.8	7.5	1
*FAT3*	6.8	7.0	1
*ZFHX4*	6.8	8.4	1
*KDM6A*	4.5	4.8	1
*NFE2L2*	4.5	5.3	1
*TRIO*	4.5	5.3	1
*MYH4*	2.3	4.0	1
*TNN*	2.3	1.3	0.510
*FBXW7*	0	3.5	0.361

*Fisher’s exact test, ^†^Chi-square tests.

ADGRV1, adhesion G protein-coupled receptor V1; CDKN2A, cyclin dependent kinase inhibitor 2A; CSMD3, CUB and Sushi multiple domains 3; DMD, dystrophin; DNAH5, dynein axonemal heavy chain 5; EP300, E1A binding protein p300; FAT1, FAT atypical cadherin 1; FAT3, FAT atypical cadherin 3; FBXW7, F-box and WD repeat domain containing 7; FSIP2, fibrous sheath interacting protein 2; HMCN1, hemicentin 1; KDM6A, lysine demethylase 6A; KMT2D, lysine methyltransferase 2D; LRP1B, LDL receptor related protein 1B; LRP2, LDL receptor related protein 2; MUC16, mucin 16, cell surface associated; MUC17, mucin 17, cell surface associated; MYH4, myosin heavy chain 4; NFE2L2, nuclear factor, erythroid 2 like 2; NOTCH1, notch receptor 1; PCLO, piccolo presynaptic cytomatrix protein; PIK3CA, phosphatidylinositol-4,5-bisphosphate 3-kinase catalytic subunit alpha; RB1, RB transcriptional corepressor 1; SI, sucrase-isomaltase; SYNE1, spectrin repeat containing nuclear envelope protein 1; TNN, tenascin N; TP53, tumor protein p53; TRIO, trio Rho guanine nucleotide exchange factor; TTN, titin; USH2A, usherin; XIRP2, xin actin binding repeat containing 2; ZFHX4, zinc finger homeobox 4.

## Discussion

Using a combination of a 35-gene mutation profile, clinical nodal staging, and RT dosimetry, mutations in *FSIP2* and *SYNE1* were identified as potential predictors of outcomes of definitive CCRT in patients with ESCC.

Conventional clinicopathological factors and RT dosimetry parameters have been found to be correlated with prognosis in terms of the tumor spreading extent and RT toxicity to the lung. A previous report indicated that in patients with esophageal cancer treated with CCRT and concurrent CCRT, the radiation pneumonitis rate was significantly increased when lung V30 ≥13% (10), and dosimetric variables, including lung V30 >8%, were associated with worse OS in univariate analysis (11), respectively. Therefore, we included lung V30 ≥10% in the analysis model and found that it was a predominant prognostic factor compared with the T and N stages, for PFS and OS. Radiation-induced lung injury including radiation pneumonitis and pulmonary fibrosis are major, sometimes fatal, dose-limiting toxicities of thoracic RT, which may affect the prognosis of patients (12). Regarding the intrinsic characteristics of tumors, gene mutations or mutation profiles examined using the NGS panel revealed a distinct correlation between the clinical outcomes of definitive CCRT. *FSIP2*, located at 2q32.1, encodes a fibrous sheath-interacting protein. The fibrous sheath is a cytoskeletal structure in the sperm flagellum (13). Recurrent amplification of *FSIP2* has been reported in 22% of seminomas (14) and 15.3% of testicular germ cell tumors (15). A higher *FSIP2* mutation rate was reported in metastatic breast cancer compared to that in early stage breast cancer (16). Additionally, FSIP2 shows high expression in patients with clear cell renal cell carcinoma and is associated with poor survival outcomes and prognosis (17). Collectively, these results indicate that FSIP2 plays a role in metastasis, tumor invasion, and chemotherapeutic resistance in cancer. A mutation may cause the loss of FSIP2 expression and therefore act as a favorable PFS marker for ESCC. *SYNE1* and forkhead box protein E1 promoter methylation have been identified as candidate biomarkers in colorectal cancer plasma DNA (18). A high promoter hypermethylation rate of up to 80% was detected in the biopsy samples of patients with colitis-associated colorectal cancer (19). In addition, cumulative evidences suggest that changes in SYNE1 expression levels, somatic mutations, promoter methylation level, and single-nucleotide polymorphisms are related to the occurrence and development of lung cancer (20), oral cancer (21), hepatocellular carcinoma (22), and gastric cancer (23). Furthermore, *SYNE1* was found to be frequently mutated in an Indian ESCC cohort (24). In the present study, we reported *SYNE1* mutations associated with worse prognosis in patients with ESCC, which is consistent with a previous report of patients with clear cell renal cell carcinoma showing that *SYNE1* mutations correlate with a higher tumor mutation burden and poorer outcomes (25). Additionally, SYNE1 mutations in patients with clear cell renal cell carcinoma are involved in immune response signal and alterations based on the profiles of infiltrating immune cells (25). As radiation is known to trigger the immunologic response, SYNE1 mutation may involve the radioresistant signal. Our results showed that mutations in *FSIP2* and *SYNE1* have opposite effects on the survival of patients with ESCC treated with definitive CCRT. Further investigations are needed to explore the role of *FSIP2* and *SYNE1* mutations in the development of biomarkers or treatment targets.

The mutation rates of several genes differed between our ESCC cohort and TCGA cohort (26, 27). As two TCGA cohorts comprised patients from southern and northern China, the different gene mutation rates may not be due to ethnic difference. However, the frequency of locally advanced stage (stage III and IV) in our cohort (86.4%) was significantly higher than in TCGA cohort (50.2%), which may have led to the different mutation rates. It is unclear whether the different mutation rates were related to the tobacco smoking status, consumption of alcoholic beverages, and exposure to fine particulate matters (such as PM_2.5_) or indoor air pollutants (such as polycyclic aromatic hydrocarbons) (2, 28). A previous study of a Chinese cohort reported no significant differences in the rate or composition of mutations between smokers and non-smokers and suggested that smoking contributes to the ESCC risk *via* mechanisms distinct from those in other smoking-related cancers (29). Different allele frequency thresholds of mutations in targeted genes, disease etiology, or disease stage in different studies also cause differences in the mutation rate. These observations indicate that the gene mutation profiles among different sources have significant variations; hence, NGS data should be interpreted with caution.

A limitation of this study was the lack of germline mutation data for comparison with somatic mutations to identify the actual somatic mutations. In addition, the number of samples, particularly those from females, used for NGS was relatively small; therefore, the reported mutation frequencies may not be fully representative of a larger population. Inclusion of adequate numbers of male and female patients with ESCC is required in further studies. Mutant genes may generate chemoresistant or radioresistant tumor cells and alter the chemosensitivity or radiosensitivity of patients with ESCC. Further validation of the biological functions and clinical roles of *FSIP2* and *SYNE1* in both ESCC experimental models and patients is warranted.

In conclusion, a combination of a 35-gene mutation profile and RT dosimetry identified mutations in *FSIP2* and *SYNE1* as well as lung V30 and clinical nodal staging as potential predictors for developing a prediction model for clinical outcomes of patients with ESCC treated with definitive CCRT.

## Data Availability Statement

The datasets presented in this study can be found in online repositories. The names of the repository/repositories and accession number(s) can be found below: https://www.ncbi.nlm.nih.gov/sra/PRJNA742478.

## Ethics Statement

The studies involving human participants were reviewed and approved by Ethics Committee of Tianjin Medical University Institute and Hospital (Documentation number #bc2018057). The patients/participants provided their written informed consent to participate in this study. Written informed consent was obtained from the individual(s) for the publication of any potentially identifiable images or data included in this article.

## Author Contributions

Y-JC conceived and supervised all works. Y-CH and Y-JC designed, analyzed and drafted the article. PT, CT, QP, YW and ZY collected the patient samples and clinical data. C-WC participated interpretation the data. All authors contributed to the article and approved the submitted version.

## Funding

This research was funded in part by research grants from the MacKay Memorial Hospital, Taipei, Taiwan (MMH-E-109-13 and MMH-E-110-13), Ministry of Science and Technology, Taiwan (MOST 108-2320-B-039-023-MY3), and China Medical University, Taiwan (CMU109-MF-22).

## Conflict of Interest

The authors declare that the research was conducted in the absence of any commercial or financial relationships that could be construed as a potential conflict of interest.

## Publisher’s Note

All claims expressed in this article are solely those of the authors and do not necessarily represent those of their affiliated organizations, or those of the publisher, the editors and the reviewers. Any product that may be evaluated in this article, or claim that may be made by its manufacturer, is not guaranteed or endorsed by the publisher.
